# GLP-1 receptor agonist liraglutide has a neuroprotective effect on an aged rat model of Wolfram syndrome

**DOI:** 10.1038/s41598-019-52295-2

**Published:** 2019-10-31

**Authors:** Kadri Seppa, Maarja Toots, Riin Reimets, Toomas Jagomäe, Tuuliki Koppel, Maia Pallase, Stine Hasselholt, Maiken Krogsbæk Mikkelsen, Jens Randel Nyengaard, Eero Vasar, Anton Terasmaa, Mario Plaas

**Affiliations:** 1 Institute of Biomedicine and Translational Medicine, Department of Physiology, University of Tartu, 19 Ravila Street, Tartu, 50411 Estonia; 2 Institute of Biomedicine and Translational Medicine, Laboratory Animal Centre, University of Tartu, 14B Ravila Street, Tartu, 50411 Estonia; 3 Centre of Excellence for Genomics and Translational Medicine, University of Tartu, Ravila 19, Tartu, 50411 Estonia; 4 Core Center for Molecular Morphology, Section for Stereology and Microscopy, Department of Clinical Medicine, Aarhus University, Aarhus, Denmark

**Keywords:** Endocrine system and metabolic diseases, Neurodegeneration, Molecular medicine

## Abstract

Wolfram syndrome (WS) is a rare neurodegenerative disorder that is mainly characterized by diabetes mellitus, optic nerve atrophy, deafness, and progressive brainstem degeneration. Treatment with GLP-1 receptor agonists has shown a promising anti-diabetic effect in WS treatment in both animal models and in human patients. Since previous research has tended to focus on investigation of the WS first symptom, diabetes mellitus, the aim of the present study was to examine liraglutide effect on WS-associated neurodegeneration. We took 9-month-old Wfs1 knock-out (KO) animals that already had developed glucose intolerance and treated them with liraglutide for 6 months. Our research results indicate that 6-month liraglutide treatment reduced neuroinflammation and ameliorated endoplasmic reticulum (ER) stress in the inferior olive of the aged WS rat model. Liraglutide treatment also protected retinal ganglion cells from cell death and optic nerve axons from degeneration. According to this, the results of the present study provide novel insight that GLP-1 receptor agonist liraglutide has a neuroprotective effect in the WS rat model.

## Introduction

One rare neurodegenerative disorder with an autosomal recessive inheritance pattern is Wolfram syndrome (WS), which is caused by biallelic mutations of the Wolframin ER transmembrane glycoprotein (Wfs1) gene and first manifests as diabetes mellitus, followed by optic nerve atrophy, deafness, and symptoms of neurodegeneration^1–3^. WS patients have symptoms of ataxia, sleep apnea, dysphagia, hearing loss, and loss of taste due to brainstem atrophy; therefore, progressive neurodegeneration can be considered as a widespread feature of WS^4,5^. Currently, there is no experimentally proven cure for WS-accompanying neurodegeneration, and death usually occurs in the fourth decade due to brain stem atrophy-induced respiratory failure^5^. Therefore, it is most important to develop effective neuroprotective therapies that are able to slow the progression of the disease and extend the lives of patients.

Our research group created and validated a Wfs1 transgenic rat with exon 5 disruption. As a result of dysfunctional Wfs1 protein, all the main symptoms found in human were present in the Wfs1 transgenic rat, including endoplasmatic reticulum (ER) stress in the medulla, increased extraparenchymal space (EPS) around the medulla, and decreased medullary volume, with the largest decreases at the level of the inferior olive^6^. The inferior olive (IO), which consists of the medial nucleus, dorsal nucleus and principal nucleus, is essential to the development of fine motor control and coordination^7^. Degeneration of the inferior olive has also been reported in human WS patients^4^. The brainstem neurodegeneration pattern in Wfs1 transgenic rats shows similarities to the neurodegeneration observed in human WS patients. Therefore, Wfs1 transgenic rats are a valuable model for translational research connected to neurodegeneration.

Recently, our group has demonstrated in Wfs1 knock-out (KO) rats that 19-week-long treatment with Glucagon-like-peptide-1 (GLP-1) receptor agonist liraglutide decreases ER stress, inflammation, and proliferation in Langerhans islets and thereby prevents or delays the development of a diabetic phenotype^8^. Moreover, treatment with the GLP-1 receptor agonist exenatide has shown a promising anti-diabetic effect in Wfs1 knock-out mice both after acute^9^ and in chronic administration^10^.

Accumulating evidence suggests that GLP-1 receptor agonists have additional roles other than glucose-lowering effects. Expression of the GLP-1 receptor is widely detected in various cells besides pancreatic beta-cells, such as in neurons, astrocytes, and microglia, suggesting that GLP-1 might have a neuroprotective role^11^. Through receptor autoradiography of rat brain tissue sections, the highest densities of GLP‐1 binding sites in the brainstem have been identified in the area postrema (AP), the nucleus of the solitary tract (NTS), and in the inferior olive^12^. Likewise to GLP-1 receptor, Wfs1 is also expressed in the inferior olive^6^. Therefore, we investigate here if late intervention with GLP-1 receptor agonist liraglutide has a neuroprotective effect and thereby can prevent the progression of inferior olive neurodegeneration in the rat model of WS.

## Results

### 6-month treatment with the GLP-1 receptor agonist liraglutide delayed the progression of hyperglycemia

**Figure 1:**
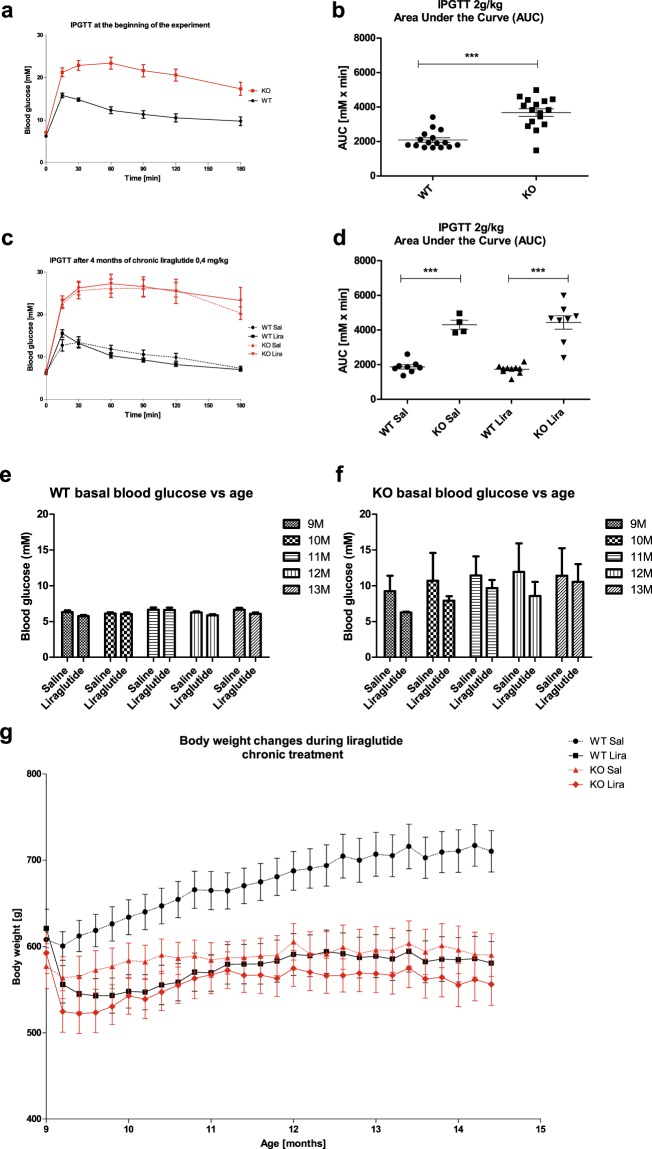
Characterization of diabetic phenotype and body weight during the liraglutide treatment period. For intraperitoneal glucose tolerance tests (IPGTTs), blood glucose levels were measured after administration of glucose (2 g/kg i.p.), and the area under the curve was calculated: (**a,b**) IPGTT at the beginning of the experiment, (**c,d**) IPGTT after 4 months of chronic liraglutide treatment. For ethical reasons, IPGTT was not performed on animals who had already developed hyperglycemia. (**e,f**) Basal blood glucose levels at different ages. (**g**) Body weight change over 6 months of liraglutide chronic treatment. The data were compared using factorial ANOVA followed by Fisher’s LSD tests; ***p < 0.001. The data are presented as the mean ± SEM, n = 4–16 per group.

The Wfs1 KO rats were already glucose intolerant at the beginning of the experiment (Fig. 1a,b) (F(1, 30) = 38.69, p < 0.001 (genotype)), which agrees with our previous studies^6,8^. As expected, 13-month-old KO animals were still glucose intolerant (Fig. 1c,d) (F(1,26) = 103.50, p < 0.001 (genotype)) but, surprisingly, 6-month treatment with the GLP-1 receptor agonist liraglutide delayed the progression of hyperglycemia in Wfs1 KO rats mostly during the first months of treatment (Fig. 1f) (F(1,149) = 27.63, p < 0.001 (genotype); F(1,149) = 3.96, p < 0.05 (treatment)).

After one week of treatment, liraglutide induced a decrease in body weight in both genotypes, whereas wild-type (WT) littermates from the vehicle group continued to grow (Fig. 1g) (F(1,168) = 24.46, p < 0.001 (genotype); F(1,168) = 31.77, p < 0.001 (treatment); F(1,168) = 10.16, p < 0.01 (genotype × treatment)).

### Medullary volume increased with age

**Figure 2:**
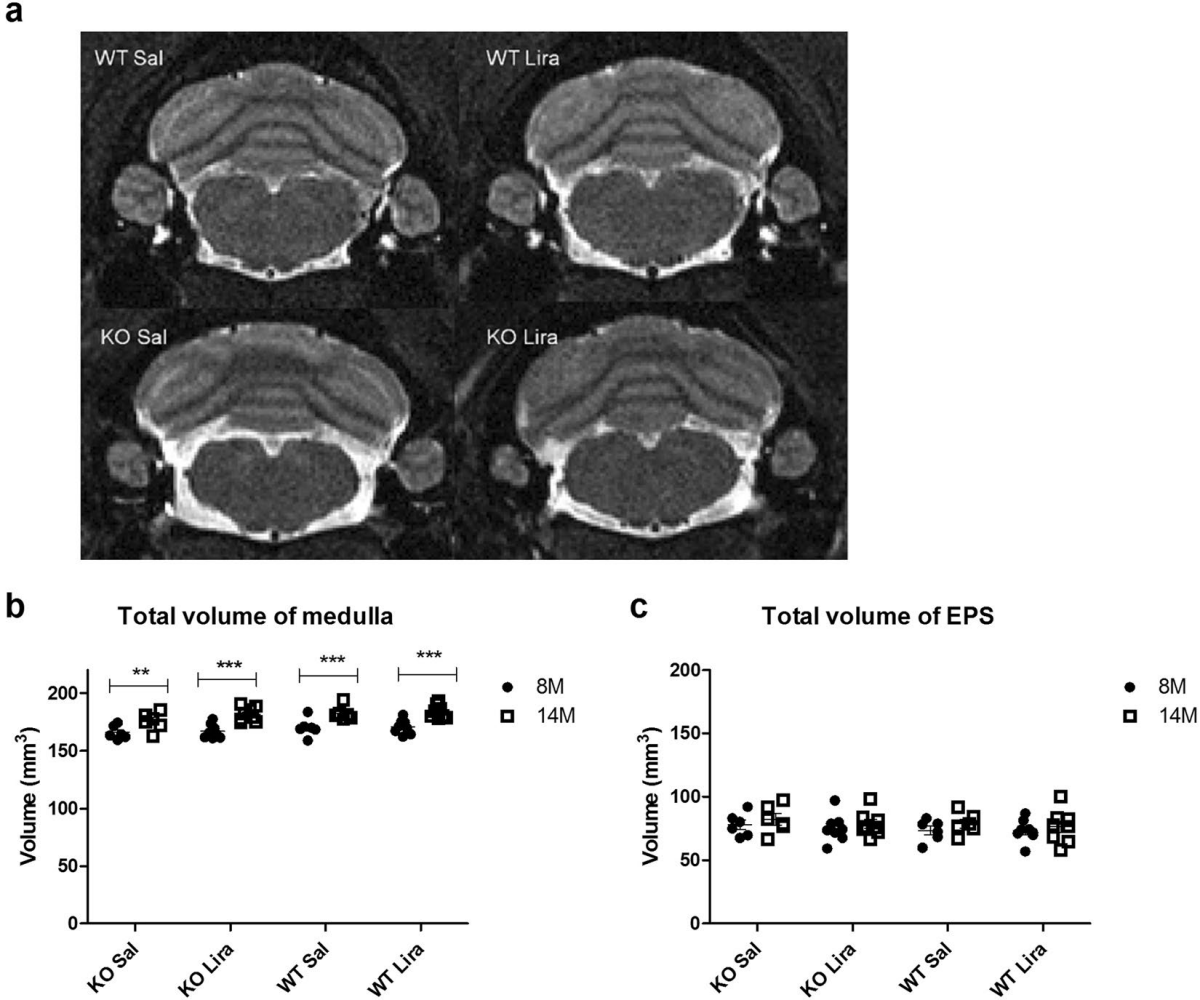
Medullary volume is increased with age in all experimental groups. (**a**) Representative T2-weighted MR images of the medulla of saline-treated wild-type (WT) and Wfs1 knock-out (KO), liraglutide-treated WT and Wfs1 KO rats are taken at the level of the inferior olive (bregma approx. −12.63 mm). Quantitative MRI analysis of (**b**) medullary volume and (**c**) extraparenchymal space (EPS) were manually traced by an observer blinded to the genotypes of the rats from T2 images using ITK-SNAP software. The volumes of the segmented structures were calculated as volume per slice from bregma level −9.48 to −15.48 mm. The data were compared using repeated measures ANOVA followed by Bonferroni post hoc tests; **p < 0.01, ***p < 0.001. The data are presented as the mean ± SEM, n = 6–10 per group.

First, we performed Magnetic Resonance Imaging (MRI) analyses to measure the medullary anatomical changes during liraglutide treatment (Fig. 2a). T2-weighted *in vivo* MRI analysis revealed that medullary volume increased with age in all experimental groups (Fig. 2b) (F(1,24) = 125.12, p < 0.0001 (age). There was also an age effect in EPS (F(1,24) = 7.91, p < 0.05 (age) (Fig. 2c).

### After liraglutide treatment, there was an increased number of neurons in Wfs1 KO animals’ dorsal nuclei

To determine neurodegeneration, we used stereological quantification to estimate the total number of neurons in the inferior olive. Stereology was performed under the assumption that the volume of the three nuclei of the Inferior olive was unchanged between the groups. This is confirmed by the fact that the total volume of the medulla was unchanged between different experimental groups at the age of 14 months (Fig. 2b), and the volume of all inferior olive subnuclei was unchanged (Supplemetary Fig. 2).

**Figure 3:**
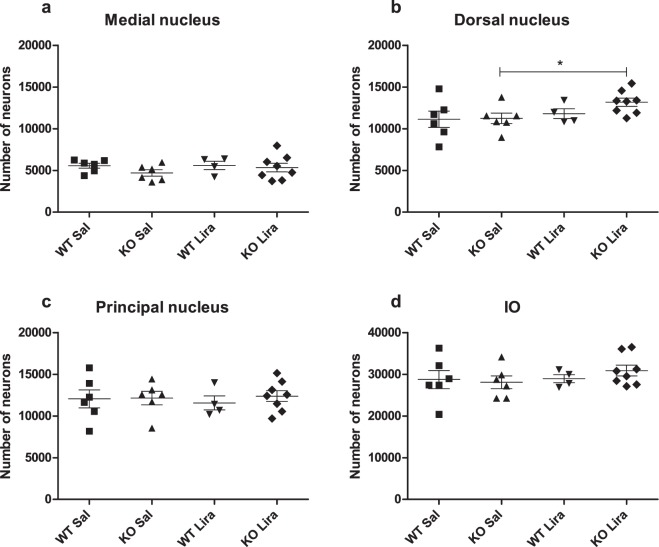
After liraglutide treatment, there was an increased number of neurons in Wfs1 KO animals’ dorsal nucleus. Stereological quantification of the total number of neurons in the inferior olive after 6 months of liraglutide treatment. Total number of neurons in (**a**) medial nucleus, (**b**) dorsal nucleus, (**c**) principal nucleus, and (**d**) in all inferior olive (IO) nuclei together. The data were compared using factorial ANOVA followed by Fisher’s LSD tests; *p < 0.05. The data are presented as the mean ± SEM, n = 4–8 per group.

Thionin staining effectively stained neurons (Supplemetary Fig. 1). The total number of neurons in the inferior olive was estimated in three major subnuclei in the inferior olive—in the medial nucleus, dorsal nucleus, and principal nucleus—using the optical fractionator (Supplemetary Fig. 1). In the dorsal nucleus, there were an increased number of neurons in liraglutide-treated KO animals. In addition, there was a tendency to overall treatment effect in the dorsal nucleus (Fig. 3b) (F(1,20) = 1.06, p = 0.31 (genotype); F(1,20) = 3.29, p = 0.08 (treatment); F(1,20) = 0.78, p = 0.39 (genotype × treatment)). There were no significant changes in other inferior olive subnuclei: no difference in the medial nucleus (Fig. 3a) (F(1,20) = 1.41, p = 0.25 (genotype); F(1,20) = 0.54, p = 0.47 (treatment); F(1,20) = 0.43, p = 0.52 (genotype × treatment)) in the principal nucleus (Fig. 3c) (F(1,20) = 0.27, p = 0.61 (genotype); F(1,20) = 0.02, p = 0.89 (treatment); F(1,20) = 0.17, p = 0.68 (genotype × treatment)); or in all inferior olive nuclei together (Fig. 3d) (F(1,20) = 0.14, p = 0.71 (genotype); F(1,20) = 0.83, p = 0.37 (treatment); F(1,20) = 0.61, p = 0.44 (genotype × treatment)).

### Neuronal volume increased in Wfs1 KO animals

**Figure 4:**
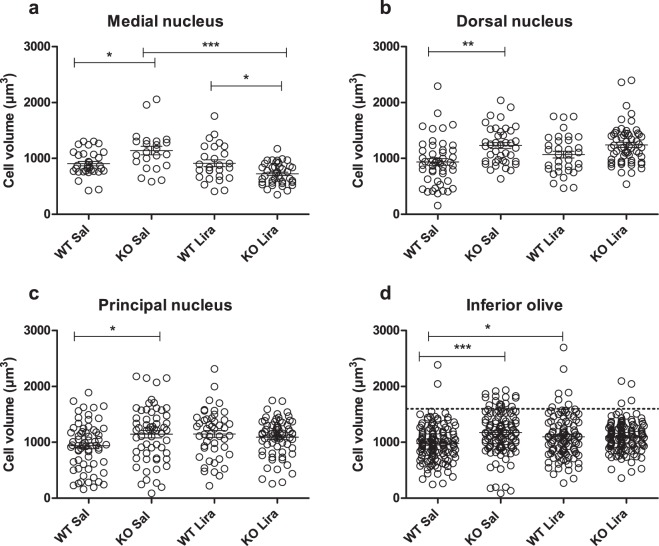
Neuronal volume is increased in Wfs1 KO animals. Neuronal cell volume in (**a**) medial nucleus; (**b**) dorsal nucleus; (**c**) principal nucleus; and (**d**) in all inferior olive (IO) nuclei together. There is an increased neuronal volume in saline-treated Wfs1 KO animals compared to WT littermates in all subnuclei, suggesting neuronal swelling in Wfs1 KO animals. (**a**) Liraglutide treatment decreased neuronal swelling in the medial nucleus in Wfs1 KO animals. (**d**) In Wfs1 KO animals there is an increased number of large neurons (1600 µm^3^ and above, dotted line), as compared to liraglutide-treated Wfs1 KO animals, indicating that liraglutide treatment prevented neurons from swelling. The data were compared using one-way ANOVA followed by Tukey’s HSD tests; *p < 0.05, **p < 0.01, ***p < 0.001. The data are presented as the mean ± SEM, n = 4–7 per group.

Next, we measured the soma volume of individual neurons using the spatial rotator. Neuronal volume was increased in Wfs1 KO rats compared to WT littermates in all subnuclei (Tukey’s HSD posthoc test, Fig. 4), indicating possible neuronal swelling in Wfs1 KO animals. Liraglutide treatment decreased neuronal swelling in the medial nucleus in Wfs1 KO animals (Fig. 4a). In all inferior olive nuclei together (Fig. 4d), there was an increased volume of neurons in liraglutide-treated WT animals.

### Liraglutide treatment decreased ER stress in the inferior olive

**Figure 5:**
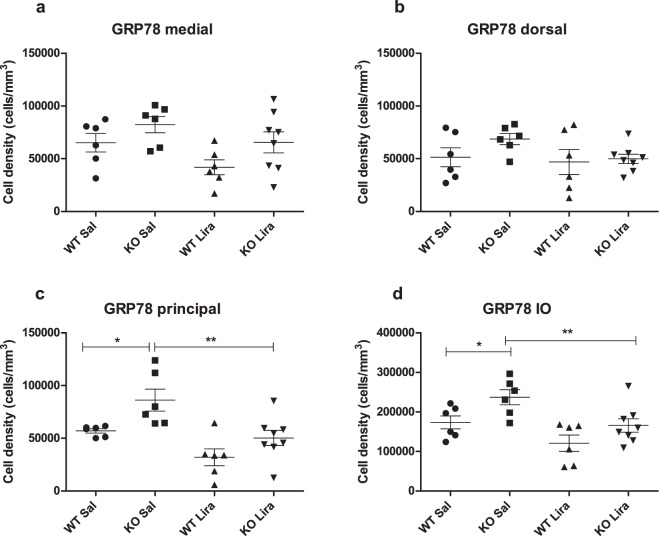
Liraglutide treatment decreased ER stress in the inferior olive. Stereological quantification of cell density of GRP78-positive neurons in the inferior olive after 6 months of liraglutide treatment in (**a**) medial nucleus; (**b**) dorsal nucleus; (**c**) principal nucleus; and (**d**) in all inferior olive nuclei together. The data were compared using factorial ANOVA followed by Fisher’s LSD tests; *p < 0.05, **p < 0.01. The data are presented as the mean ± SEM, n = 6–8 per group.

As neuronal cell volume alterations may refer to a pathological response, we performed a stereological estimate of ER stress markers to assess the overall health of neuronal cells in the inferior olive. GRP78-positive cells were visualized by staining with anti-GRP78 antibody. The staining effectively labeled cells in every treatment group and genotype (Supplementary Fig. 3). Stereological estimation was performed using the optical disector estimate neuron densities. Due to the fact that the volume of the different nuclei in the inferior olive was unchanged between groups (Supplementary Fig. 2), differences in neuron density can be regarded as changes in their total number. The density of ER stress marker GRP78-positive cells was increased in Wfs1 KO rats compared to WT littermates in all inferior olive nuclei together (Fig. 5d) (F(1,22) = 8.62, p < 0.01 (genotype)). Liraglutide treatment decreased ER stress in KO animals inferior olive (F(1,22) = 11.30, p < 0.01 (treatment); F(1,22) = 0.25, p = 0.62 (genotype × treatment)), resulting in similar levels in WT and liraglutide-treated KO animals. Factorial ANOVA showed significant differences in the cell density of GRP78-positive cells in the medial nucleus (Fig. 5a) (F(1,22) = 5.25, p < 0.05 (genotype); F(1,22) = 5.06, p < 0.05 (treatment); F(1,22) = 0.14, p = 0.71 (genotype × treatment)) and also there were significant differences in the cell density in the principal nucleus (Fig. 5b) (F(1,22) = 9.61, p < 0.05 (genotype); F(1,22) = 15.79, p < 0.01 (treatment); F(1,22) = 0.50, p = 0.48 (genotype × treatment)). No significant differences were found in the dorsal nucleus (Fig. 5b) (F(1,22) = 1.69, p = 0.21 (genotype); F(1,22) = 2.23, p = 0.15 (treatment); F(1,22) = 0.85, p = 0.38 (genotype × treatment)).

### Liraglutide treatment decreased neuroinflammation in the inferior olive

**Figure 6:**
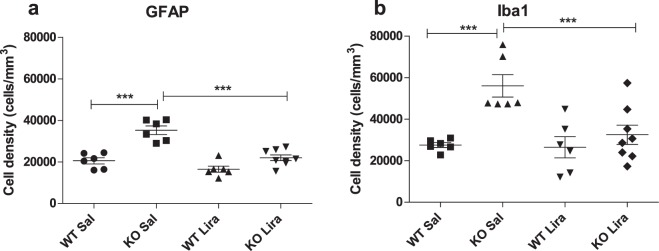
Liraglutide treatment decreased neuroinflammation in the inferior olive. Stereological quantification of GFAP- and Iba1-positive cells in the inferior olive after 6 months of liraglutide treatment, (**a**) GFAP-positive astroglia and (**b**) Iba1-positive microglia in the inferior olive. The data were compared using factorial ANOVA followed by Fisher’s LSD tests, ***p < 0.001. The data are presented as the mean ± SEM, n = 6–8 per group.

Our next purpose was to assess the inflammatory response in the inferior olive. This was performed using the optical disector to estimate Ionized calcium-binding adapter molecule 1 (IBA1)-positive microglia cells and Glial fibrillary acidic protein (GFAP)-positive astroglia cell densities (Fig. 6).

IBA1-positive cells were visualized by staining with anti-IBA1 antibody, and the staining effectively labeled cells in every treatment group and genotype (Supplementary Fig. 4). Significant increase in Iba1-positive microglia cells was detected in Wfs1 KO rats compared to WT littermates in all inferior olive nuclei together (Fig. 6b) (F(1,22) = 14.50, p < 0.001 (genotype)). Liraglutide treatment suppressed microglial activation in KO animals (Fig. 6b) (F (1,22) = 7.35, p < 0.05 (treatment); F(1,22) = 6.14, p < 0.05 (genotype × treatment)).

GFAP-positive cells were visualized by staining with anti-GFAP antibody, and the staining effectively labeled cells in every treatment group and genotype (Supplementary Fig. 5). In line with the microglial cell number, the number of GFAP-positive astrocytes was substantially elevated in the inferior olive of KO animals compared to WT littermate controls (Fig. 6a) (F(1,22) = 39.80, p < 0.001 (genotype). Liraglutide treatment decreased GFAP-positive activated astrocytes F(1,22) = 28.97, p < 0.001 (treatment); F(1,22) = 8.16, p < 0.01 (genotype × treatment)).

### Liraglutide treatment protected from retinal ganglion cell death

**Figure 7:**
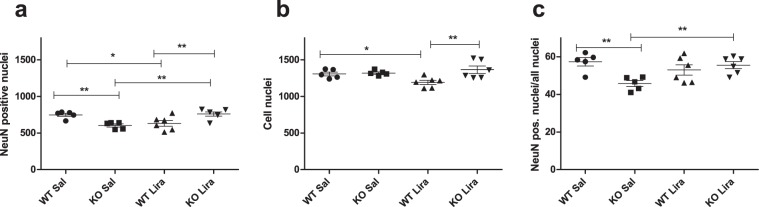
Liraglutide treatment protected from retinal ganglion cell death. Cell nuclei quantification in retinas after 6 months of liraglutide treatment: (**a**) Quantification of NeuN positive nuclei, (**b**) quantification of all nuclei stained with Hoechst H33258, (**c**) quantification of NeuN positive nuclei/all nuclei after 6 months of liraglutide treatment. The data were compared using factorial ANOVA followed by Fisher’s LSD tests; * p < 0.05, **p < 0.01. The data are presented as the mean ± SEM, n = 5–6 per group.

Next, we measured whether liraglutide treatment has an effect on the retinal ganglion cell number. Neuronal nuclei were stained with NeuN and all nuclei with H33258, and the staining effectively labeled cells in every treatment group and genotype (Supplementary Fig. 6). The ratio of pan-neuronal marker (NeuN)-positive nuclei to total nuclei showed a considerable decrease in KO animals compared to WT littermate controls (Fig. 7c) (F(1,18) = 4.167, p = 0.0562 (genotype)). Liraglutide treatment normalized the ratio in KO rats and had no effect on WT animals (F(1,18) = 10.18, p < 0.01 (genotype × treatment)).

### Liraglutide treatment protected Wfs1 KO rats’ optic nerve axons from degeneration

**Figure 8:**
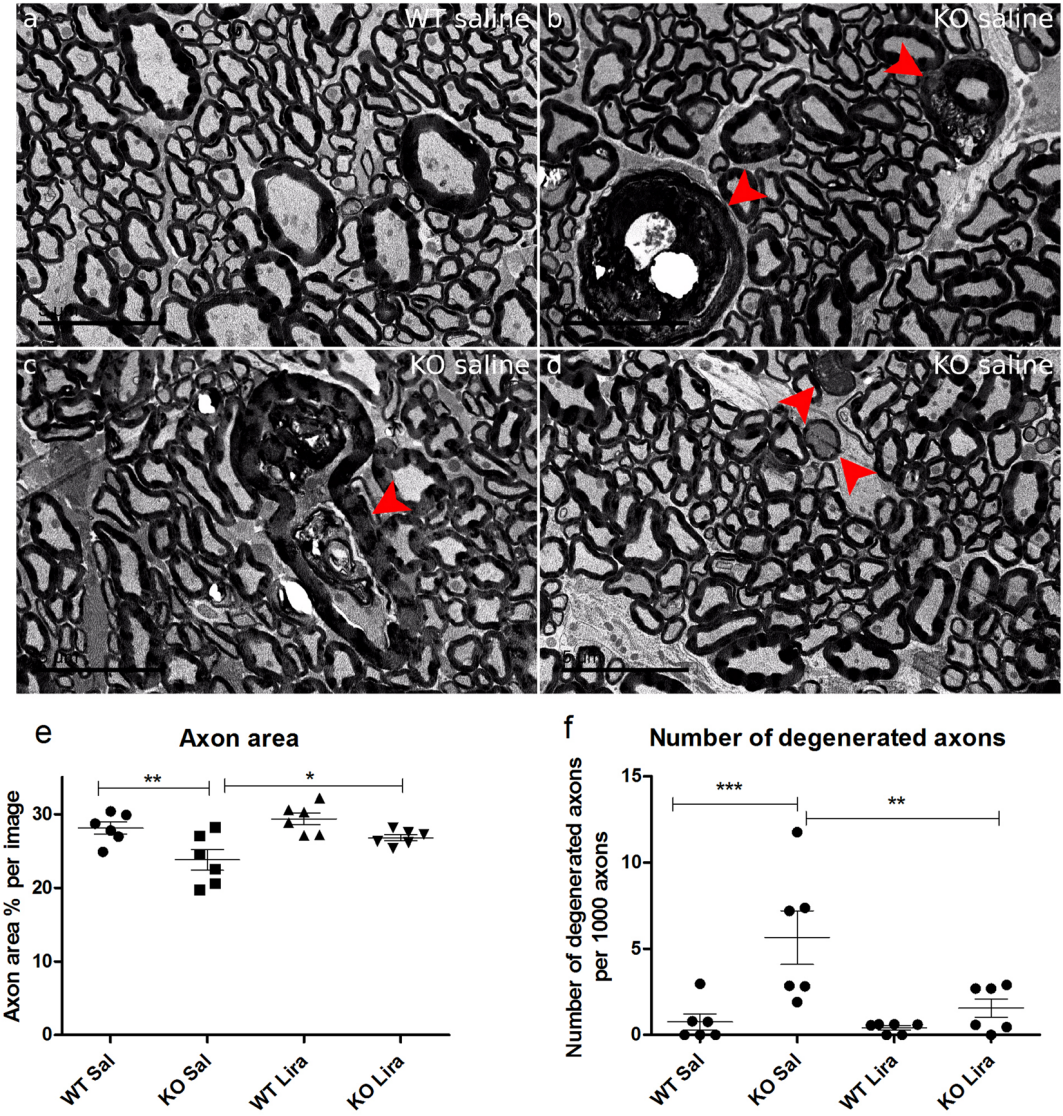
Liraglutide treatment protected Wfs1 KO rats’ optic nerve axons from degeneration. Effect of 6 months of liraglutide treatment on Wfs1 KO rat optic nerve. Representative electron micrographs of (**a**) WT Sal and (**b–d**) KO Sal rats. Optic nerves of KO Sal rats display several features of axon degeneration resulting in (**b,c**) myelin collapse (arrowheads), (**d**) extensive vacuolization and electrondense axoplasm (arrowheads). (**e**) Liraglutide treatment protected KO animals from axonal area decline. Axon area per animal in percentage was compared. (**f**) Liraglutide treatment protected KO animals from axonal degeneration, measured as number of degenerated axons per 1000 axons. The data were compared using factorial ANOVA followed by Fisher’s LSD tests; *p < 0.05, **p < 0.01, ***p < 0.001. Scalebar: 5 μm. The data are presented as the mean ± SEM, n = 6 per group.

The percentage of optic nerve axons area per area was decreased in saline treated Wfs1 KO animals compared to WT saline-treated littermates (Fig. 8e) (F(1,20) = 13.317, p < 0.01 (genotype). Liraglutide treatment prevented a decrease in axon area in Wfs1 KO animals (F(1,20) = 5.140, p < 0.05 (treatment)). Next, myelin collapse as a result of axon degeneration was observed from transmission electron microscopy images. Saline-treated Wfs1 KO rats had a significantly higher number of degenerated axons per 1000 axons than WT rats (Fig. 8f) (F(1,20) = 12.43, p < 0.01 (genotype)). Liraglutide treatment protected KO animals from axonal degeneration and had no effect on WT rats (F(1,20) = 6.72, p < 0.05 (treatment); F(1,20) = 4.76, p < 0.05 (genotype × treatment)).

## Discussion

This study provides the first evidence of the feasibility of pharmacological treatment for life-threatening neurodegeneration in an aged WS rat model.

Usually, WS is diagnosed after the onset of metabolic syndrome and the appearance of the first neurodegenerative symptoms, such as loss of vision. For that reason, it is important to find not only antidiabetic treatments but also therapies that could delay the progression of neurodegeneration and thereby extend the quality of life and lifespan of WS patients. We have shown that the GLP-1 receptor agonist liraglutide has the ability to slow down the progression of the diabetic phenotype in our validated Wfs1-deficient rat model^8^. Wfs1 and GLP-1 receptor are both expressed in the inferior olive and in the retina, and therefore treatment with the GLP-1 receptor agonist may have a direct neuroprotective effect on those structures^6,12–14^. The present study was designed to investigate whether late intervention with liraglutide has a neuroprotective effect on an aged rat model of WS.

In the current study, to mimic the relatively late diagnosis of WS in patients we used 9-month-old Wfs1 KO animals that already had developed glucose intolerance (Fig. 1a,b). As we have shown previously, at the age of 7 months, Langerhans islets start to decline, and, at the age of 14 months, the majority of Langerhans islets have disappeared^6^. Therefore, a 6-month treatment with liraglutide in this study did not improve glucose tolerance, as expected, and 13-month-old animals were still glucose intolerant (Fig. 1c,d), although a 6-month treatment with the GLP-1 receptor agonist liraglutide delayed the progression of hyperglycemia in Wfs1 KO rats, mostly during the first months of treatment (Fig. 1f). It can be assumed that the remaining beta cells were protected by liraglutide treatment, and thereby beta cell degeneration was delayed. Alternatively, reduced base blood sugar could be partly due to weight loss, since, after a one-week treatment, liraglutide induced a decrease in body weight in both genotypes, whereas WT littermates continued to grow (Fig. 1g). These results are in agreement with our previous study, in which we also saw weight loss after the first week of liraglutide treatment^8^. Our present results indicate that starting the treatment after the development of metabolic syndrome could not restore glucose tolerance, which was expected. Nevertheless, our main aim was to investigate whether the liraglutide treatment could still have a therapeutic effect on neurodegeneration.

A recent study showed that WS patients have reduced brainstem and cerebellar volume, and it is suggested that the Wfs1 mutation could affect axonal myelination over time^15^. Here we also monitored changes in animals’ medullary volume over time. The results of T2-weighted *in vivo* MRI analysis at the level of the inferior olive revealed an age effect in the total volume of the medulla after six months of treatment (Fig. 2b). Medullary volume is increased with age in all experimental groups.

Brainstem atrophy and accompanying degeneration of the inferior olive is a recognized feature of WS^1,4,16,17^. Post mortem neuropathological examinations have revealed neuronal loss and gliosis in WS patients’ brainstems^4,18^, although it is not known when the loss of neurons starts. We used stereology to determine whether the number of neurons had changed in 14-month-old animals and if liraglutide treatment had effect on it. Our results indicate that the total number of neurons changed in the inferior olive dorsal nucleus; specifically, liraglutide-treated Wfs1 KO animals had an increased number of neurons compared to saline-treated Wfs1 KO animals (Fig. 3b).

Next, we measured the soma volume of individual neurons using the spatial rotator. Neuronal volume increased in Wfs1 KO rats compared to WT littermates (Fig. 4). The increase in neuronal volume in Wfs1 KO rats can be defined by neuronal swelling, whereby extracellular Na^+^ and other cations enter into neurons and accumulate intracellularly, resulting in an influx of anions and water, which in turn results in osmotic expansion of the cell. Axonal swelling has also been shown in human WS patients^15^. Neuronal swelling is a cytopathological response which may include glutamate receptor activation and an inhibition of Na^+^/K^+^-ATPase^19^. It has previously been shown that knockdown of Wfs1 in neurons increased vulnerability to excitotoxicity-induced neurodegeneration^20^. Therefore, neuronal swelling might be due to WS-related neuropathology caused by excitotoxicity. Liraglutide treatment protected neurons from swelling in Wfs1 KO rats, and thus it can be suggested that liraglutide provides neuroprotection against excitotoxicity. Previously, it has been shown that liraglutide has the ability to rescue neuronal cells from oxidative stress and glutamate excitotoxicity-induced cell death^21^. This could be one possible mechanism of how liraglutide also exerts its neuroprotective effects in the WS rat model.

Recent evidence indicates that protein misfolding and aggregation leading to ER stress is a major cause of neurodegeneration in ageing and in different neurodegenerative diseases such as Alzheimer’s disease (AD), Parkinson’s disease (PD), Amyotrophic Lateral Sclerosis (ALS), and HD^22^. Also, in WS it is believed that WFS1 deficiency leads to impairment of cellular calcium regulation and an increase in ER stress and, as a consequence, causes cell death^5^. The ER chaperone GRP78 (Endoplasmic reticulum chaperone BiP) is used as a marker of ER stress due to its ability to control the activation of transmembrane ER stress sensors Inositol-requiring enzyme 1 (IRE1), Protein kinase RNA-like endoplasmic reticulum kinase (PERK), and Activating transcription factor 6 (ATF6) through a binding-release mechanism^22^. Using a stereological quantification, we found an increased number of GRP78-positive cells in Wfs1 KO animals’ inferior olive, and our results indicate that liraglutide treatment reduced ER stress in Wfs1 KO animals’ inferior olive (Fig. 5). This finding is in agreement with our previous data, namely that increased GRP78 levels have been detected in Wfs1 KO rats’ pancreases and in the ventral medulla^6,8^. Additionally, we have previously shown that liraglutide treatment reduced GRP78 levels in Langerhans islets^8^. Furthermore, our present results indicated that starting the treatment after the development of metabolic syndrome could not restore glucose tolerance and hence insulin secretion. This finding supports the idea that the neuroprotective effect of liraglutide is not dependent on the improvement of the metabolic phenotype. Thus, liraglutide treatment appears to be insulin-independent and have similar ER stress-lowering effects on both beta cells and on neurons in a rat model of WS. This is also supported by the normalization of GRP78 levels, which is the same in beta cells^8^ and neurons (Fig. 5). The similarity of cellular stress response in beta cells and in neurons highlights the value of the Wfs1 KO rat as a pre-clinical tool not only to find treatment strategies for diabetes mellitus but also for ER stress-associated neurodegenerative diseases.

Activation of microglia is a widespread feature of neurodegeneration that appears in different neurodegenerative diseases^23^. Using a stereological quantification, we found a significant increase in Iba1 positive microglia cells in Wfs1 KO rats (Fig. 6b). The inflammation response, as measured by activated microglia, was halved in liraglutide-treated Wfs1 KO rats. The ability of GLP-1 receptor agonists to suppress cytotoxic microglial responses and enhance cytoprotective phenotypes has demonstrated benefits across different experimental models^24^, such as in models of stroke^25^, Alzheimer’s disease^26^, Parkinson’s disease^27^, and traumatic brain injury^28^.

In line with microglia response, the number of GFAP-positive astrocytes was substantially elevated in KO animals compared to WT littermate controls, and liraglutide treatment decreased GFAP-positive astrocytes similarly to microglia activation (Fig. 6a). GFAP is considered a marker protein for astrogliosis, which has been implicated in the pathogenesis of a variety of neurodegenerative diseases: Alzheimer’s disease, inflammatory demyelinating diseases, human immunodeficiency virus (HIV)-associated dementia, acute traumatic brain injury, and prion-associated spongiform encephalopathies^29^.

Previously, we have revealed clear degenerative processes in 15-month-old Wfs1 KO rats’ optic nerves and resultant atrophy^6^. Similar to previous results, we also saw here an increase in axonal degeneration in Wfs1 KO rats (Fig. 8). The reduction in the area of the axons and the increase in the number of degenerated axons in Wfs1 KO animals’ optic nerves refers to neuropathological processes in which some axons are eliminated and some degenerate, which may happen prior to disappearance. Liraglutide treatment protected Wfs1 KO rats’ optic nerve axons from degeneration. Comparing the ratio of NeuN positive nuclei to total nuclei, liraglutide treatment protected Wfs1 KO rats from retinal ganglion cell death (Fig. 7c), bringing the number of neurons to a similar level with WT animals. Liraglutide’s ability to protect from axonal degeneration and retinal ganglion cell death indicates a promising neuroprotective effect.

A common obstacle for successful development of new treatment strategies for chronic diseases has been an incomplete representation of human pathologies in animal models, especially in regard to neurodegeneration^30^. The Wfs1 KO rat fully mirrors human WS pathology on the anatomical level^6^ and on the cellular level, including neuronal swelling (Fig. 4), ER stress (Fig. 5) and microglia and astroglia activation (Fig. 6) in the neurons. Importantly, degenerative processes observed in Wfs1 KO rats develop naturally in a similar way to how they develop in human patients. This is in contrast with most models of neurodegeneration, in which degenerative processes are induced artificially and do not represent human pathology to the full extent^30^. Thus, this study further supports the high translational value of the Wfs1 KO rat in neurodegeneration-related research.

In summary, this is the first study showing a neuroprotective effect in connection with WS. Long-term liraglutide treatment for aged Wfs1-deficient rats reduced neuronal inflammation and ameliorated ER stress in the inferior olive. Moreover, such treatment also prevented axonal degeneration and retinal ganglion cell death. Altogether, the results of the current study suggest that liraglutide is a promising agent to not only treat WS-associated diabetes mellitus but also to ameliorate WS-related neurodegeneration. These results show that treatment with liraglutide has a neuroprotective effect in Wfs1-deficient rats and thus may have a similar effect in WS patients.

## Materials and Methods

### Animals

Generation and phenotype of a Wfs1 mutant (Wfs1 exon 5 knock-out) rat has been described previously^6^. Breeding and genotyping were performed at the Laboratory Animal Centre at the University of Tartu. For this study, male homozygous Wfs1-deficient and WT littermate control rats were used. The animals were housed in cages in groups of 2–4 animals per cage under a 12-h light/dark cycle (lights on at 7 a.m.). Rats had unlimited access to food and water except during testing. Sniff universal mouse and rat maintenance diet (Sniff #V1534) and reverse osmosis-purified water were used. Experiments were performed between 9 a.m. and 5 p.m.

All experimental protocols were approved by the Estonian Project Authorisation Committee for Animal Experiments (No 103, 22nd of May 2017), and all experiments were performed in accordance with the European Communities Directive of September 2010 (2010/63/EU).

### Intraperitoneal glucose tolerance tests (IPGTT)

Animals were deprived of food for 3 h before and during the experiment; water was available throughout the experiment. D-Glucose (Sigma-Aldrich) was dissolved in 0.9% saline solution (20% w/vol) and administered intraperitoneally at a dose of 2 g/kg of body weight. Blood glucose levels were measured at the indicated time points from the tail vein using a handheld glucometer (Accu-Check Go, Roche, Germany). Blood samples were drawn from the tail vein immediately before and 30 min after glucose administration for further analyses. In IPGTT after 4 months of treatment, the sample size is smaller in the Wfs1 KO Saline group because two animals had developed hyperglycemia, and, therefore, they were excluded from the IPGTT.

### Chronic liraglutide treatment

The rats were 8 months old at the beginning of the experiment. Rats were randomly allocated into the liraglutide or control group (WT Sal, n = 8; WT Lira, n = 10; KO Sal, n = 6; KO Lira, n = 8). The liraglutide group animals received 0.4 mg/kg liraglutide (Novo Nordisk, Denmark), and the control group animals received a 0.9% saline solution (vehicle) subcutaneously for 6 months. Injections of 1 ml/kg volume were made once a day between 8 and 11 a.m. Rats were weighed once a week, and their base blood sugar level was measured once a month from the tail vein using a handheld glucometer (Accu-Check Go, Roche, Germany).

### Data analysis

The data were analysed using Statistica version 8 software (Statistica, Tulsa, OK, USA) or GraphPad Prism version 5 software (GraphPad Software Inc., San Diego, CA, USA). p < 0.05 was considered statistically significant. The data are presented as the mean ± SEM and were compared using factorial ANOVA followed by Fisher’s LSD tests or one-way ANOVA followed by Tukey’s HSD tests. Significance was measured between genotype and treatment; WT Saline vs KO Saline, WT Liraglutide and KO Liraglutide, KO Saline vs KO Liraglutide and WT Saline vs WT Liraglutide. In some experiments, the sample size is smaller because of tissue limitations, not because of the removal of data from the analysis.

### *In vivo* magnetic resonance imaging

Rats at 8 and 14 months of age were anaesthetized using isoflurane (1.5–2.5% in 1.5 l/min medical oxygen) and placed on a heated animal bed throughout the MRI procedure. All scans were performed using a 9.4 T Bruker BioSpec 94/20 USR system connected to a 1 H circular polarized transceiver coil and running ParaVision 6.0.1® software (Bruker BioSpin Group, Bruker Corporations, Germany). Respiration and temperature were monitored using a respiration pillow and a rectal probe (SA Instruments Inc., Stony Brook, USA). Respiration rate was maintained at 35–50 breaths per minute. Two orientation pilot scans were performed in order to establish the position of the animal and identify anatomical landmarks relevant for planning the subsequent scan. The final T2-weighted Turbo RARE sequence was performed using the following parameters: repetition time (TR) 6803 ms, echo time (TE) 33 ms, flip angle 90 degrees, number of averages 5, imaging matrix 320 × 320 × 65, spatial resolution 0.16 × 0.16 × 0.5 mm.

Volumes were segmented manually by an observer blinded to the genotype using ITK-SNAP (V3.6.0)^31^. For the medulla, EPS and cerebellum segmentation began from the caudal end of the inferior colliculus and continued until reaching the most caudal level of the cerebellum (bregma −9.48 to −15.48 mm).

### Tissue preparation

Rats were anaesthetized with an intraperitoneal injection of ketamine (100 mg/kg) and dexmedetomidine (20 mg/kg) in a volume of 10 ml/kg and were then perfused transcardially with 300 ml phosphate buffered saline (PBS) and thereafter with 300 ml 4% paraformaldehyde (PFA, Sigma-Aldrich) in a 0.1 M phosphate buffer (PB, pH 7.4). Brains were dissected and further fixed in the same fixative overnight at 4 °C. Tissues were placed in a 30% sucrose (AppliChem)/0.1 M PB solution until they sank and were then frozen at −80 °C until further use.

Brainstems were sectioned, 50 μm coronally from caudal to rostral, bregma level −13.30 to −11.80 mm (Paxinos and Watson, 2014) on a Microm HM355 cryostat (Microm International GmbH, Walldorf, Germany). Systematic uniform series with a random start were saved in cryoprotectant solution.

### Immunohistochemistry of the brainstem

Sections were washed (3 × 5 min) in Tris buffered saline (TBS) containing 0.3% Triton X-100 (TBST buffer), endogenous peroxidases were deactivated by incubating in peroxidase block (hydrogen peroxide) for 10 min with gentle agitation, and sections were rinsed with TBS and incubated with target retrieval solution (Dako, Glostrup, Denmark) for 30 minutes at 80 °C and washed again with TBST (3 × 5 min). Sections were blocked with blocking solution containing 10% goat serum/1% bovine serum albumin (BSA, Sigma-Aldrich)/TBST or 1% bovine serum albumin (BSA, Sigma-Aldrich)/TBST for 1 h at room temperature. Primary and secondary antibodies were diluted in 1% BSA/TBST. Sections were incubated with primary antibodies overnight at 4 °C with gentle agitation and were then washed with TBST (3 × 15 min). Sections were incubated with the appropriate secondary antibody at room temperature for 2 h. After subsequent washes with TBS (3 × 15 min), sections were placed in solution for DAB reaction (Sigma–Aldrich) (10 min). Sections were washed with TBS (3 × 10 min), mounted on SuperFrost glass slides (MenzelGlaser) using 0.5% Gelatine (Sigma–Aldrich) + 0.05% Chromalum (BDH Chemicals Ltd) dissolved in dH_2_O, dried at room temperature for 20 min, rehydrated in dH_2_O for 15 min, and dehydrated in a graded series of ethanol solutions (2 min in 96%, 5 min in 99%). Finally, sections were cleared for 3 × 5 min in xylene, and cover slips (No. 0, Hounisen) were mounted using Eukitt Quick-hardening mounting medium (Sigma–Aldrich).

Primary antibodies and their dilutions were as follows: rabbit anti-GRP78 antibody (Abcam ab31390; 1:2000), goat anti-Iba1 antibody (Abcam ab107159; 1:1000), rabbit anti-GFAP antibody (Synaptic Systems ab887720; 1:1000), rabbit anti-XBP1 alpha antibody (Abcam ab6671; 1:500), and rabbit anti-ATF-4 antibody (Abcam ab31390; 1:500). Secondary antibodies and their dilutions were as follows: goat anti-rabbit (DAKO, REF P0448; 1:400), rabbit anti-goat (DAKO, REF PO449; 1:400).

For illustrative supplementary panels (Supplementary Fig. 3–5), a Leica DM750 light microscope, a Leica HI PLAN 40x lens (NA = 0.17) and a Leica ICC50 HD, controlled by Leica Application Suite v.4.6.0 software, were used (Leica Microsystems, Switzerland).

### Stereological estimate of inferior olive neuron number

Sections were stained in 0.25% thionin (CAS 78338–22–4, Sigma–Aldrich, St. Louis, Missouri, USA) for 60 sec, rinsed in dH2O for 5 sec, and dehydrated in a graded series of ethanol solutions (1 min in 70%, 2 min in 96%, 5 min in 99%). After clearing in xylene for 3 × 5 min, cover slips (No. 0, Hounisen, Skander-borg, DK) were mounted using Eukitt® Quick-hardening mounting medium (CAS 25608–33–7, Sigma–Aldrich, Steinheim, DE).

Sections were analysed using an Olympus BX51 light microscope (Olympus) equipped with a Prior motorized stage, a Heidenhain microcator, an Olympus UPlanApo 4x lens (NA = 0.16), an Olympus UPlanSApo 60x oil lens (NA = 1.35) and an Olympus DP70 digital camera controlled by newCAST (Visiopharm, Hoersholm, Denmark) software.

There are three major subnuclei in the inferior olive: the medial nucleus, dorsal nucleus and principal nucleus. Delineation of the region of interest (ROI) was performed using a 4 × objective, and the analyses were performed with a 60 × oil objective, with total magnification of 2791.36 (Supplementary Fig. 1).

The total number of neurons, N(neu), in the inferior olive was estimated using the optical fractionator^32–34^:

$$N(neu)=(\frac{1}{ssf})\cdot (\frac{1}{asf}\,)\cdot (\frac{1}{hsf})\cdot \sum {Q}^{-}(neu)$$

where ssf, asf and hsf are referred to as the section sampling fraction (1/4); the area sampling fraction (counting frame area/(dx*dy)), in which (dx*dy) indicates step length in the x- and y-direction; and height sampling fraction (h/t_Q−)_, respectively. The height is denoted h and Q^−^ weighted section thickness is denoted t_Q−_, and ∑Q^−^ (neu) is the total number of neurons counted per rat in all examined sections.

The step lengths in the x- and y-direction were 123 µm, and the area of the unbiased counting frame was 2125 µm^2^. The guard height was 5 µm and the disector height, h, was 10 µm, determined following a z-axis analysis.

Cell soma volume estimates of individual neurons were estimated using the spatial rotator^35^.

A systematic set of sections was used for the determination of cell density using the optical disector for GRP78, Iba1 and GFAP positive cells.

The numerical density of neurons N_v_ is given by^32^:

$${N}_{v}\,({\rm{c}}{\rm{e}}{\rm{l}}{\rm{l}})=\frac{{\sum }^{}{Q}^{-}(cell)}{h\,\cdot (a/p)\cdot \,{\sum }^{}P}=\frac{{\sum }^{}{Q}^{-}(cell)}{h\,\cdot (\frac{counting\,frame\,area}{p})\cdot {\sum }^{}P}$$

where p denotes the number of test points per counting frame; P denotes total number of test points hitting the ROI, and ∑Q^−^ (cell) marks the total number of cells counted per rat in all examined sections. The counting frame area was 2125 µm^2^, and height, h, was 10 µm.

### Whole-mount retina immunohistochemistry

After perfusion, eyes were removed and immersion-fixed overnight in 4% PFA/0.1 M PB at 4 °C. Followed by washes with 0.1 M PB, retinas were dissected and cryoprotected in a 30% sucrose/0.1 M PB solution and frozen at −80 °C until further use. Retinas were thawed to room temperature, washed thrice for 5 min in PBS, and permeabilized overnight in a 0.5% Triton X-100 (Naxo, Tartu, Estonia)/PBS solution at 4 °C. Subsequently, tissues were incubated in 5% normal goat serum/5% rabbit serum/1% BSA for two hours at room temperature with gentle rocking. Retinas were then incubated in primary antibody dilutions in 0.1% BSA/0.2% Tween-20/PBS for 4 days at 4 °C. Subsequently, retinas were rinsed three times in 0.1% BSA/PBS for 10 min at room temperature and then left to wash overnight at 4 °C with gentle rocking. Tissues were further incubated with the appropriate secondary antibody solutions in 0.1% BSA/0.2% Tween-20/PBS for 2 days at 4 °C and washed as previously. Nuclei were stained with 5 μg/ml Bisbenzimide H 33258 (Hoechst 33258, Sigma Aldrich) in PBS for 15 min, and retinas were rinsed with PBS for 10 min at room temperature. For imaging, retinas were placed on uncoated pre-cleaned microscope slides (Hirschmann), PBS was added, and they were covered with a 0.17-mm coverslip (Deltalab). Images from central and peripheral regions of retinas were obtained with an Olympus FV1200MPE (Olympus, Germany) laser scanning confocal microscope. Field of view with an Olympus UPlanApo 10x objective (NA = 0.4) was set to 306∙10^3^ μm^2^. H33258-stained and NeuN-positive nuclei counting was performed using ImageJ Software (version 1.52a; National Institute of Health). Threshold values for an 8-bit gray-scale image of H33258 were 10...255, and for a NeuN 8-bit gray-scale image they were 5...255. For every animal (n = 5–6 per group), at least 39000 NeuN-positive and 69000 H33258 stained nuclei were counted.

Primary antibodies and their dilutions were as follows: mouse anti-NeuN antibody (1:250, Millipore Cat# MAB377, RRID:AB_2298772), rabbit anti-GFAP antibody (1:1000, Synaptic Systems Cat# 173 002, RRID:AB_887720). Secondary antibodies and their dilutions were as follows: Rhodamine (TRITC)-AffiniPure Donkey Anti-Goat IgG (H + L) antibody (1:1000, Jackson ImmunoResearch Labs Cat# 705–025–003, RRID:AB_2340388), Donkey Anti-Mouse IgG (H + L), and Alexa Fluor®647-conjugated polyclonal antibody (1:1000, Thermo Fisher Scientific Cat# A-31571, RRID:AB_162542).

### Transmission electron microscopy

After perfusion, optic nerves were dissected and immersion fixed with 4% paraformaldehyde (AppliChem) and 2% glutaraldehyde (AppliChem) in 90 mM sodium-cacodylate buffer (CB, pH 7.4) (AppliChem) overnight at 4 °C. Optic nerves were thoroughly washed in 90 mM CB and postfixed with 1% osmium tetroxide (Electron Microscopy Sciences, EMS) in 90 mM CB. After further washes with CB, nerves were dehydrated and embedded into epoxy resin (medium hardness, TAAB). 60–90 nm sections were cut with a diamond knife (Diatome) and mounted on the PowerTome MT-XL RMC (RMC Products, Boeckeler Instruments) ultramicrotome. Analysis of specimens was performed with the Tecnai G2 Spirit BioTWIN (FEI) working at 120 kV and imaged with an Orius (Gatan) CCD camera. The number and area of axons (without the myelin sheath) were measured with ImageJ Software (version 1.52a; National Institutes of Health), where axon area per animal was measured in pixels and divided by image area in pixels (image size: 4008 × 2672 pixels); this ratio was in turn divided by the number of images per animal (n = 10–11). The resulted percentage of axon area per image was compared. Average number of axons per animal measured was from 1020 to 2208.

## Supplementary information

Supplementary information
