# Supplementary material for: GLP-1 receptor agonist liraglutide has a neuroprotective effect on an aged rat model of Wolfram syndrome
Source: Sci Rep. 2019 Oct 31;9:15742. doi: 10.1038/s41598-019-52295-2 (PMC6823542; doi:10.1038/s41598-019-52295-2)
Supplement: Supplementary file 1 — Supplementary information [file 41598_2019_52295_MOESM1_ESM.pdf]

Supplementary Material:

Title: GLP-1 receptor agonist liraglutide has a neuroprotective effect on an aged rat model of Wolfram syndrome

Authors: Kadri Seppa, Maarja Toots, Riin Reimets, Toomas Jagomäe, Tuuliki Koppel, Maia Pallase, Stine Hasselholt, Maiken Krogsbæk Mikkelsen, Jens Randel Nyengaard, Eero Vasar, Anton Terasmaa, Mario Plaas

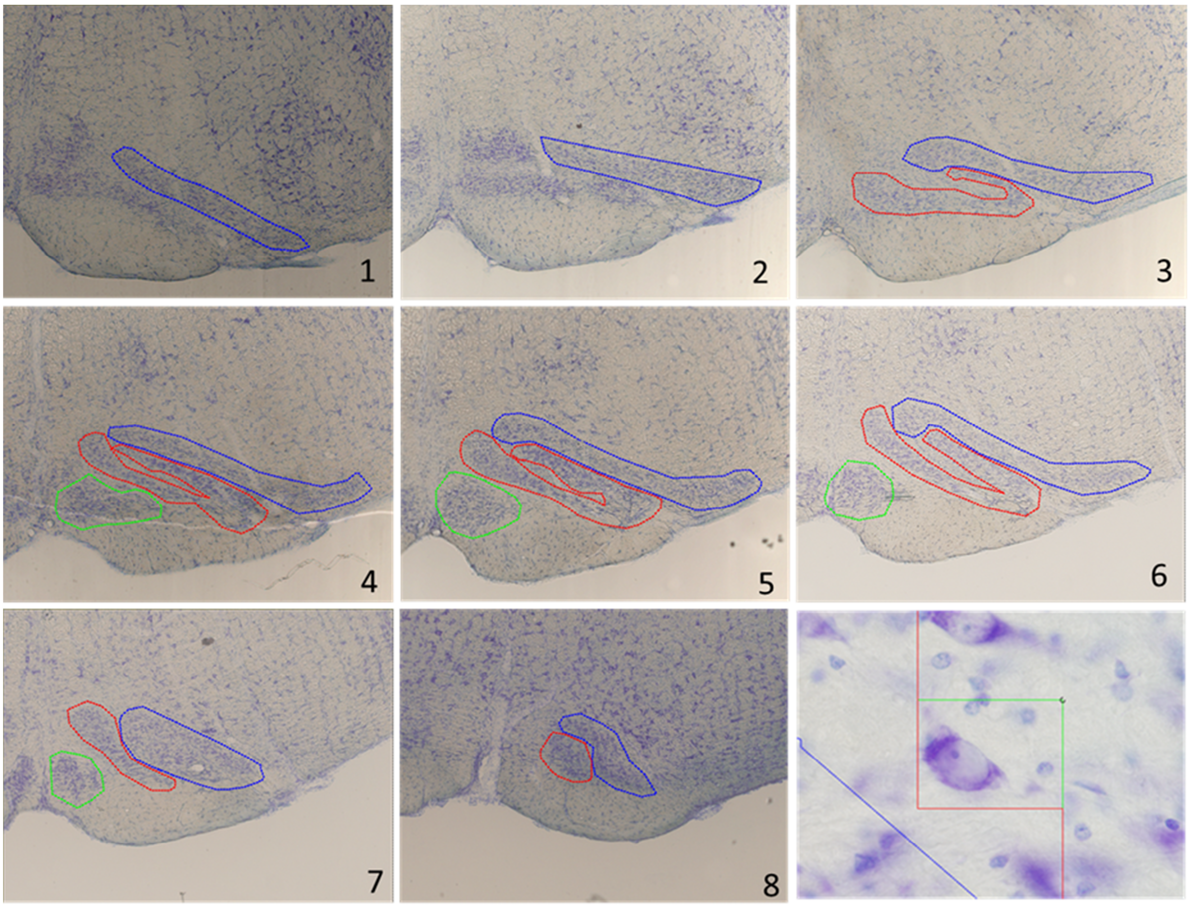

Supplementary Figure 1. The total number of neurons in the inferior olive was estimated using the optical fractionator. There are three major subnuclei in the inferior olive: the medial nucleus (green line), dorsal nucleus (blue line) and principal nucleus (red line). A systematic set of sections were Thionin stained from bregma level -12 until -14.76 and delineation of the region of interest (ROI) was performed using a 4× objective (images 1-8) and the counting was performed with a 60× oil objective (lower right image) using newCAST (Visiopharm, Hoersholm, Denmark) software.

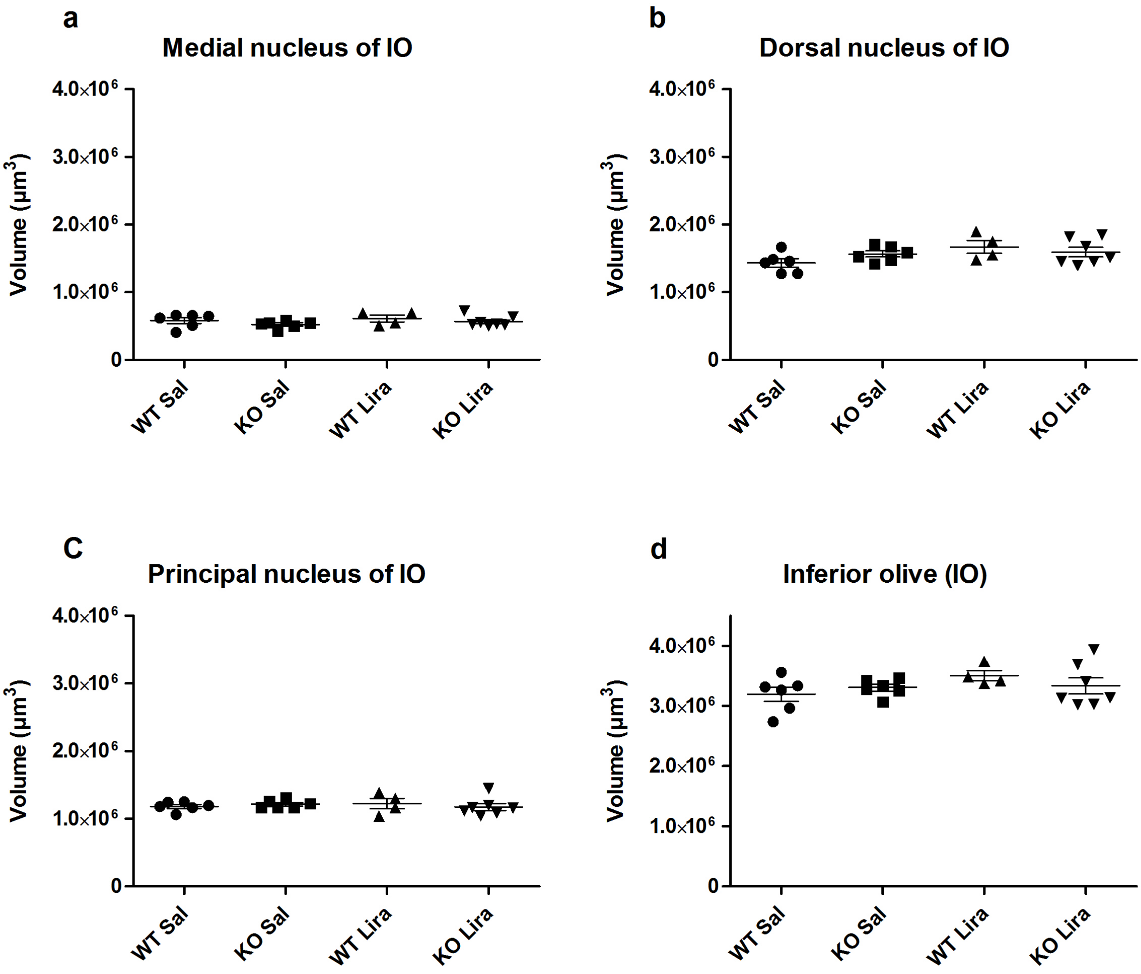

Supplementary Figure 2. Volume of inferior olive. Stereology was performed under the assumption that the volume of the three nuclei of the inferior olive was unchanged between the groups. Delineation of the region of interest (ROI) was performed using a 4× objective and the ROI volume was calculated using newCAST (Visiopharm, Hoersholm, Denmark) software.

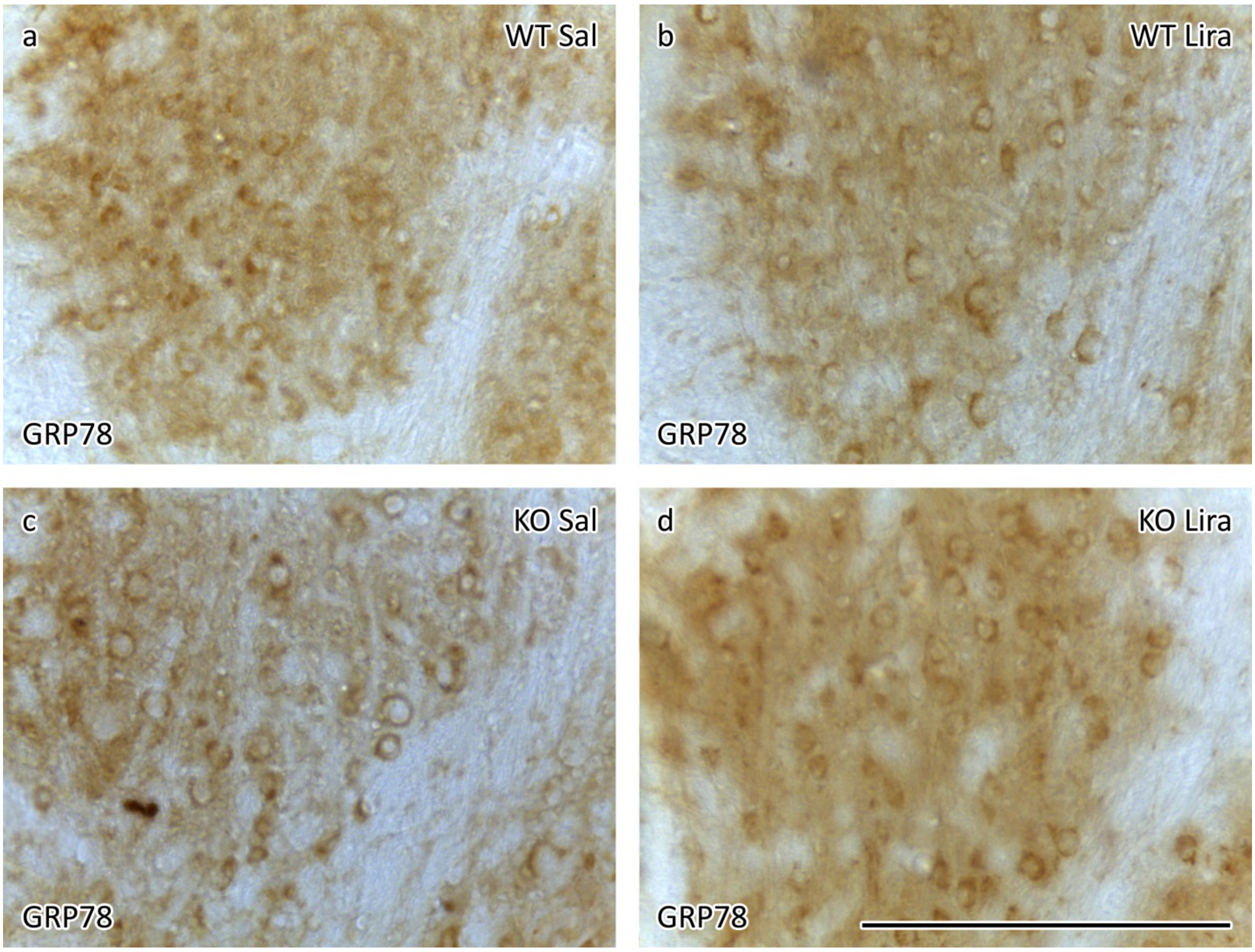

Supplementary Figure 3. GRP78 positive cells after 6 months of liraglutide/saline treatment. Staining with anti-GRP78 antibody effectively labeled neuronal cells in every treatment group and genotype (a) WT Saline, (b) WT Liraglutide, (c) KO Saline, (d) KO Liraglutide. GRP78, Endoplasmic reticulum chaperone BiP. Scale bar 200 µm.

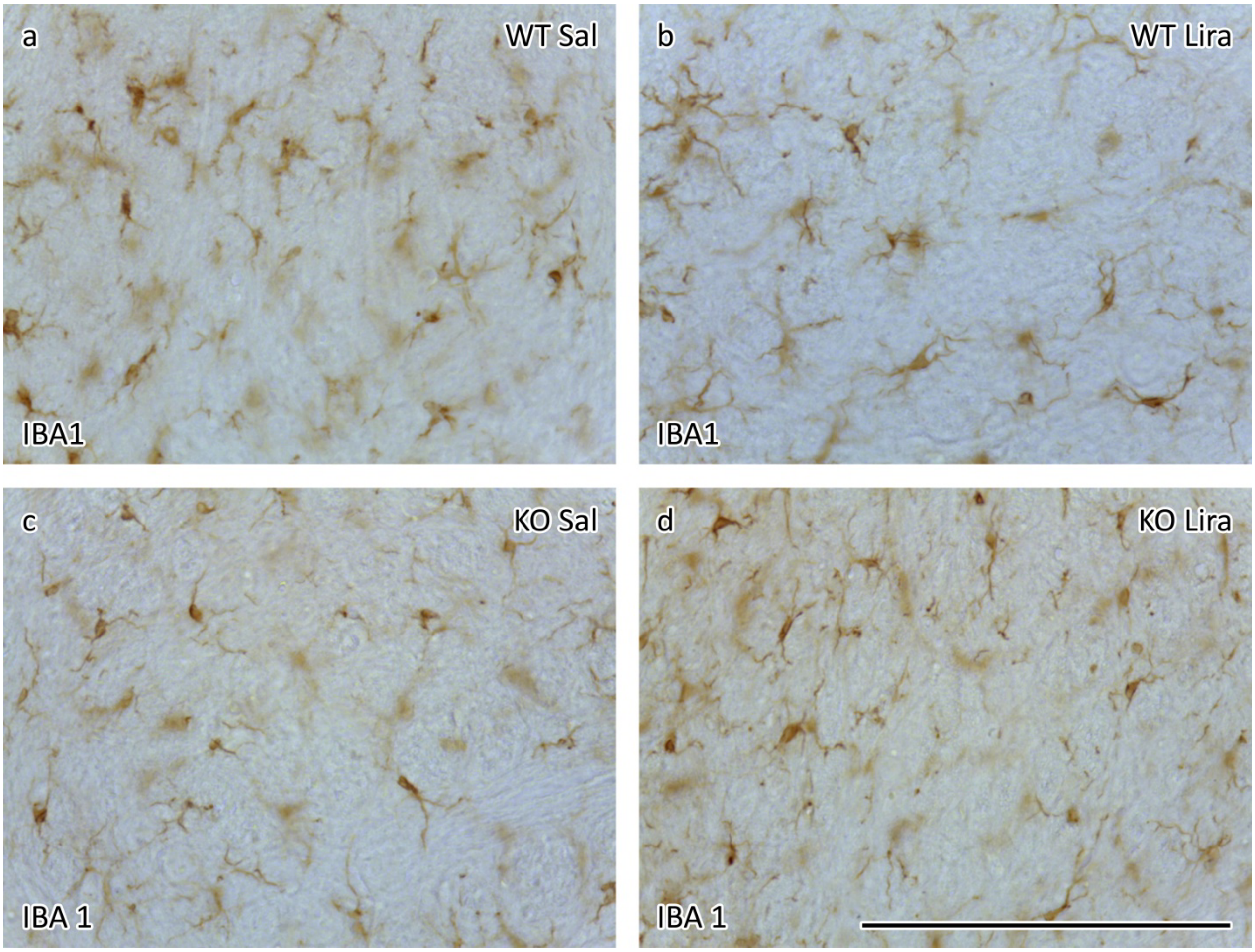

Supplementary Figure 4. Microglia cells stained with anti-IBA1 antibody after 6 months of liraglutide/saline treatment. Staining with anti-IBA1 antibody effectively labeled microglia cells in every treatment group and genotype (a) WT Saline, (b) WT Liraglutide, (c) KO Saline, (d) KO Liraglutide. IBA1, Ionized calcium-binding adapter molecule 1. Scale bar 200 µm.

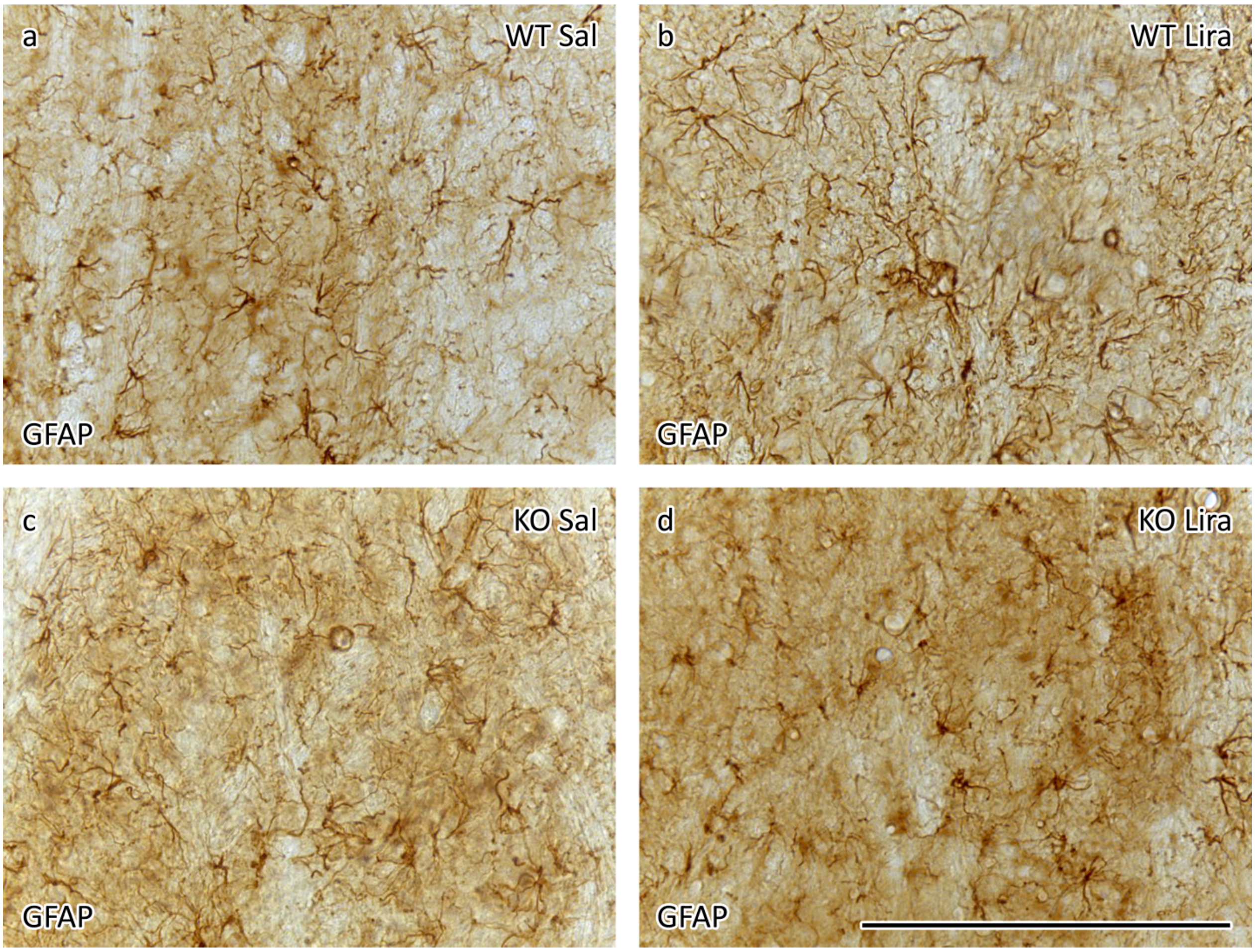

Supplementary Figure 5. Astroglia cells stained with anti-GFAP antibody after 6 months of liraglutide/saline treatment. Staining with anti-GFAP antibody effectively labeled astroglia cells in every treatment group and genotype (a) WT Saline, (b) WT Liraglutide, (c) KO Saline, (d) KO Liraglutide. GFAP, Glial fibrillary acidic protein. Scale bar 200 µm.

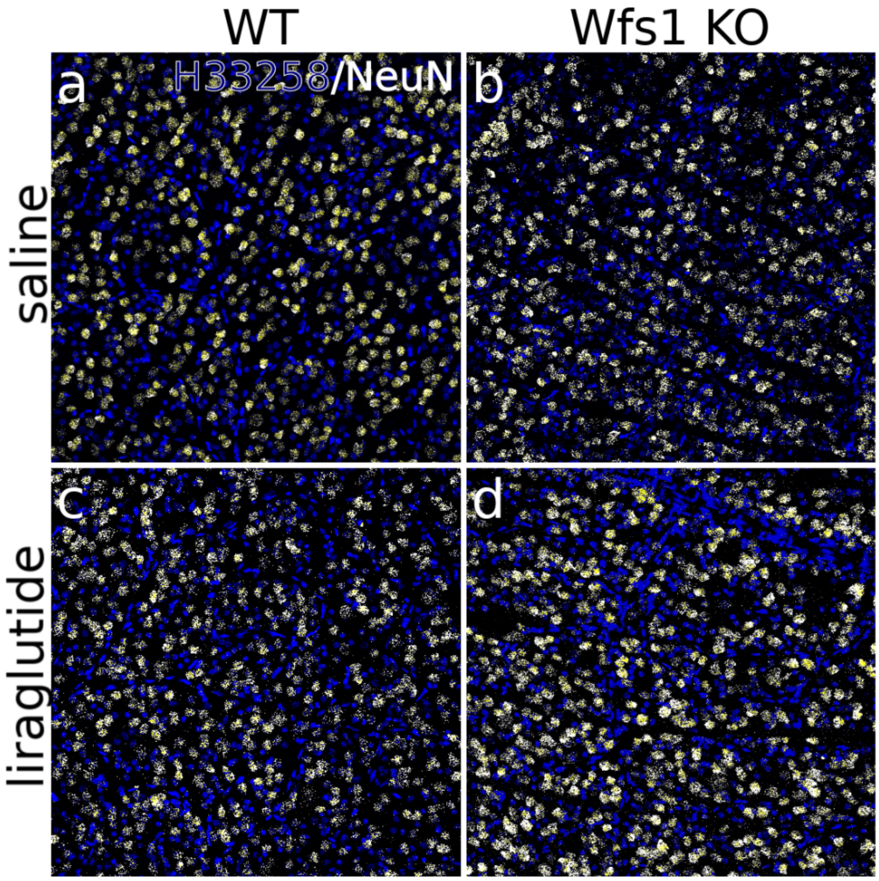

Supplementary Figure 6. Representative images of retina whole-mount immunohistochemistry. (a, b, e, f) Neuronal nuclei stained with (white) NeuN and all nuclei with (blue) H33258.
